# Study on failure mechanism on rechargeable alkaline zinc–Air battery during charge/discharge cycles at different depths of discharge

**DOI:** 10.3389/fchem.2023.1121215

**Published:** 2023-01-20

**Authors:** Donghao Zhang, Wenbin Hu

**Affiliations:** ^1^ Key Laboratory of Advanced Ceramics and Machining Technology (Ministry of Education), School of Materials Science and Engineering, Tianjin University, Tianjin, China; ^2^ Tianjin Key Laboratory of Composite and Functional Materials, School of Materials Science and Engineering, Tianjin University, Tianjin, China; ^3^ Joint School of National University of Singapore and Tianjin University, International Campus of Tianjin University, Binhai New City, Fuzhou, China

**Keywords:** rechargeable alkaline zinc-air battery, cycle life, depths of discharge, corrosion, carbonation

## Abstract

**Background:** Zinc-air battery (ZAB) is a promising candidate for energy storage, but the short cycle life severely restricts the wider practical applications. Up to date, no consensus on the dominant factors affecting ZABs cycle life was reached to help understanding how to prolong the ZAB’s cycle life. Here, a series of replacement experiments based on the ZAB were conducted to confirm the pivotal factors that influence the cycle life at different depths of discharge (DOD).

**Method:** The morphology and composition of the components of the battery were characterized by scanning electron microscopy (SEM), X-ray diffraction (XRD) and chemical titration analyses.

**Result:** SEM images and XRD results revealed that the failure of the zinc anode gradually deepens with the increase of DOD, while the performance degradation of the tricobalt tetroxide/Carbon Black (Co_3_O_4_/CB) air cathode depends on the operating time. The concentration of CO_3_^2− ^depends on the charge/discharge cycle time. The replacement experiments results show that the dominant factors affecting the ZAB’s cycle life is the reduction of active sites on the surface of Co_3_O_4_/CB air cathode at a shallow DOD, while that is the carbonation of the electrolyte at a deep DOD. The reduction of active sites on the surface of Co_3_O_4_/CB air cathode is caused by the coverage of K_2_CO_3_ precipitated by carbonation of the electrolyte, suggesting that the carbonation of the alkaline electrolyte limits ZAB’s cycle life.

**Conclusion:** Therefore, this work not only further discloses the failure mechanism of ZAB, but also provides some feasible guidance to design a ZAB with along cycle life.

## 1 Introduction

The depletion of fossil fuels and environmental degradation have led to the development of renewable energy, such as solar and wind energy, and to meet the intermittent problems of these energy production, the exploration and research of energy storage equipment is essential. Due to the limitation of cost and safety issues of traditional lithium-ion batteries, aqueous metal-air batteries have become the choice of the next-generation (Chen et al., 2022), among which Rechargeable zinc–air battery (ZAB) are most noteworthy (Wu et al., 2022) due to high energy density of 820 mA h/g which is about 5 times higher than the current lithium–ion battery (Li et al., 2013; Liu Q. et al., 2019), operate safely due to the use of non-flammable aqueous electrolyte, rich earth-abundance of Zn (Li and Dai, 2014). So it can provide stable discharge voltage for electrical vehicles, grid energy storage, even some advanced electronics, such as a robot (Goldstein et al., 1999; Yang et al., 2011; Li et al., 2013; Lutkenhaus and Flouda, 2020; Zhao et al., 2020).

Alkaline and neutral ZAB are two major research directions. Compared with milder neutral batteries, alkaline ZAB have a lower potential (−1.22 V vs. SHE) due to different negative electrode reaction processes, but the production of ZnO hinders the stability of batteries, causing the short cycle life of ZAB severely slows down the further commercialization (Shang et al., 2020). Therefore, it is important to find out the main factors affecting the cycle life and guide the improvement of ZAB’s cycle life.

The cycle life of ZAB is mainly affected by three factors: zinc anode, electrolyte, and cathode catalyst. Some researchers found Zn anode dominates ZAB’s cycle life. The work of Dongmo and his workmates (Dongmo et al., 2020) supports that hydrogen evolution reaction on the zinc anode not only damages the battery by increasing the internal pressure of the battery, but also reduces the capacity of the battery through consuming negative active materials. Thangavel et al. (Thangavel et al., 2020) stated that ZnO passivation hinders the diffusion and exchange of the reactants and products in the solution, terminating the battery reactions and leading to a shortened discharge life. Moreover, some researchers (Wang et al., 2015; Li et al., 2019; Zhang et al., 2022) claimed that Zn dendrite growth causes a short circuit between the anode and the cathode, leading to a poor rechargeability and a degradation of battery life. Meanwhile, some other researches provide different evidences that the air cathode has a great influence on the cycle life of ZAB (Dong et al., 2022). Some scholars (Sumboja et al., 2016; Min et al., 2018; Sato et al., 2020) believed the degradation of carbon-based air cathode due to the carbon corrosion during the charging progress leads to the conductivity decrease for air cathode and exacerbates the carbonation of the electrolyte, affecting the batteries’ cycle life. Catalysts suffering from poor durability and even poisoning in air cathodes limits ZAB’s performance and roundtrip efficiency (Marcus et al., 2018; Huang et al., 2019; Liu X. R. et al., 2019; Li et al., 2020; Lu et al., 2020; Zhang et al., 2020). In addition, water consumption and carbonation of electrolyte were observed to affect the cycle life of ZAB (Yang and Kim, 2019; Zhong et al., 2021). Therefore, a sufficient survey of the existing literature shows that no consensus on the dominating factors affecting the ZAB’s cycle life were reached, which severely limits the improvement of battery cycle life. Hence, there is a strong demand for the consensus on ZAB failure mechanisms to understand how to prolong the ZAB’s cycle life.

Herein, we clarify the dominate factors affecting the cycle life of ZAB at different depths of discharge (DOD). The research is expected to provide guidance for improving the cycle life of ZAB. The inconsistent results of existing studies are considered to be related to different DOD carried out by different researches. Different DOD discharges have different degrees of polarization in the battery. Generally speaking, the greater the depth of discharge, the greater the polarization of the battery, the worse the stability, and the lower the cycle life of the battery. Here, we conducted a series of charge/discharge cycle tests and replacement experiments to identify the main factors leading to the battery failure during cycling at different DOD, and then systematically analyzed each component of the cycled ZAB in depth. ZABs with a well-sealed structure were assembled at room temperature, and the components were replaced in sequence after cycling to investigate the bottleneck factor affecting the ZAB’s cycle life. To determine why the battery failed during cycling, the anode and air cathode of the cycled ZAB were characterized by X-ray diffraction (XRD) and scanning electron microscopy (SEM). The electrochemical analyses were used to study the performance change of the air cathode after cycling. In addition, chemical titration experiments were used to determine the concentration of carbonate ion (CO_3_^2–^) in the electrolytes.

## 2 Materials and methods

### 2.1 Materials

The Zn anode is made of zinc sheet, which was ground with 800/1,500 mesh sandpaper to remove the oxide layer from the surface, and then cut into 80 × 40 × 1 mm rectangular slices. Before assembly, Zn anodes were ultrasonically cleaned and dried with deionized water.

The commercial submicron-scaled Co_3_O_4_ (300 nm) powder are used as active material. The conductive additive was carbon black (CB, XC-72R) powders. The binders were polytetrafluoroethylene (PTFE).

The solution of 6 M KOH+0.2 M ZnO was fed into the electrolytic cell as the electrolyte.

### 2.2 Mold design and assembly of ZAB

**FIGURE 1:**
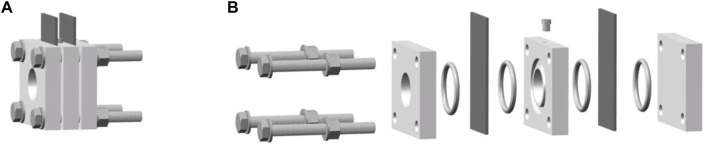
Schematic diagram of the mold of ZAB. **(A)** Schematic diagram of the assembled ZAB, **(B)** Exploded diagram of each ZAB’s component.

An easily assembled mold of ZAB with a well-sealed structure was designed with references to related research (Muller et al., 1998; Ma et al., 2014; Hong et al., 2016; An et al., 2018; Wang et al., 2018), where the distance between the air cathode and Zn anode was 1 cm, and the active area of the electrode was 4.5 cm^2^, which is illustrated as Figure 1.

Figure 1 shows a schematic diagram of the mold of ZAB. The mold comprises a support plate, an anode, an electrolytic cell, a cathode, a cover plate, and fasteners. The anode, electrolytic cell, and cathode are functional components, while the support plate, cover plate, and fasteners are auxiliary components. The support plate is used to support the ZAB. A through-hole is set in the middle of the electrolytic cell. When assembling the battery, a closed electrolytic tank is formed with Zn anode and air cathode on both sides of the electrolytic cell, which is the main area of electrochemical reactions. Meanwhile, a sampling hole is arranged on the peripheral side of the electrolytic cell to add electrolyte to the electrolytic cell and a through-hole is opened on the cover plate to supply oxygen to the air cathode. The supporting plate, electrolytic cell, and cover plate are all provided with sealing grooves to cooperate with the sealing ring to eliminate the gaps between various components. Four bolts are set at the corners of the mold to improve the mold’s sealing and avoid possible electrolyte leakage. When assembling the battery, all components are tightened by bolts in the order shown in Figure 1, and vacuum grease is applied to further eliminate the gaps between the components.

### 2.3 Cathode fabrication

**FIGURE 2:**
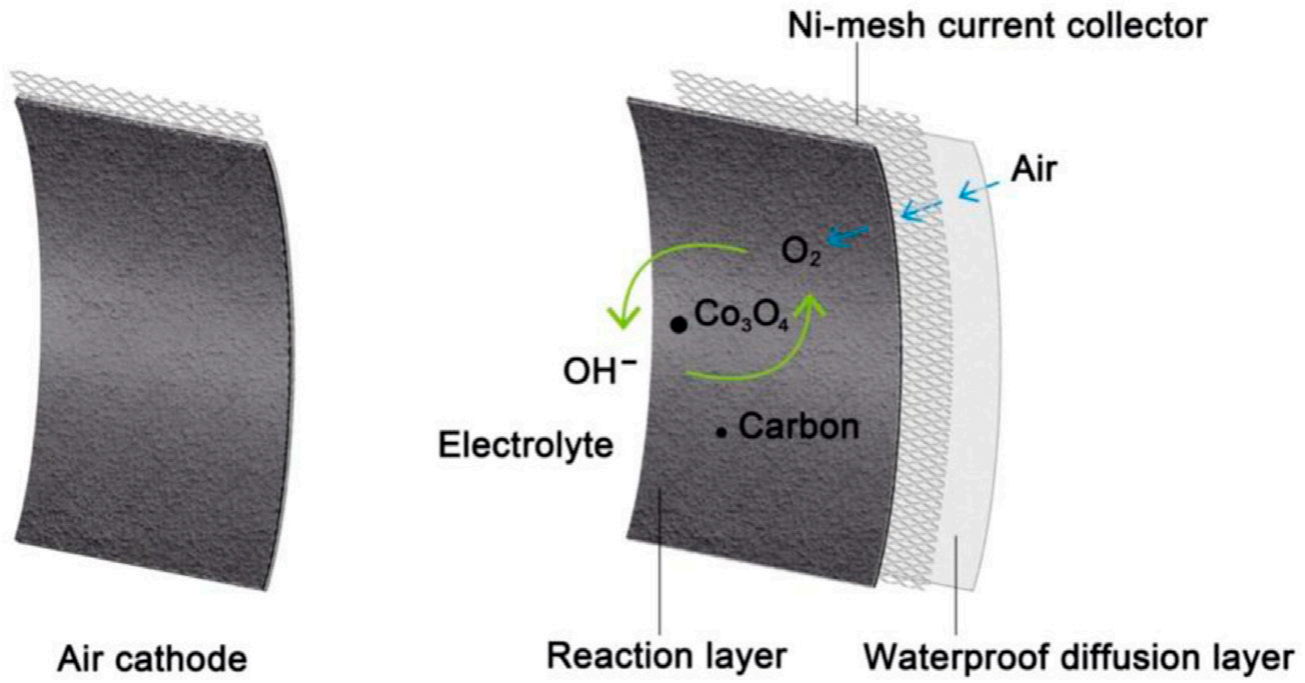
Schematic diagram of Co_3_O_4_/CB air cathode.

To fabricate the cathodes, Co_3_O_4_ active material with XC-72R was mixed with binder solution (polytetrafluoroethylene (PTFE) dissolved in ethanol) to form uniform slurries. Afterwards, the slurry was casted on a Ni-mesh current collector by a roller press to form a paste. A commercial waterproof diffusion layer was attached on the other surface of prepared cathodes. The prepared cathodes were dried at room temperature (25 °C) for 12 h, and pressed by a mechanical pressure of 20 MPa for 3 min. Finally, the electrodes were kept in vacuum oven at 60 °C for 6 h to dry completely. Then, the composition of Co_3_O_4_, XC-72R and PTFE is at a weight ratio of 30:45:25. The mass loading of Co_3_O_4_ on the Co_3_O_4_/CB air cathode is 4.5 mg cm^−2^. The structure of the prepared Co_3_O_4_/CB air cathodes is illustrated in Figure 2.

### 2.4 Performance test

The batteries were tested at room temperature (25 °C) in the atmosphere environment. No additional supply of pure oxygen was provided. First, galvanostatic discharge tests were carried out at 10 mA cm^−2^. According to the discharge capacity result, the depth of discharge (DOD) of the battery can be determined. Then, two groups of parallel experiments, deep and shallow discharge cycle tests, were carried out respectively. Last, the components were replaced in turn after the batteries had been cycled, and the cycle tests were conducted again under the original cycle conditions.

The galvanostatic discharge and charge/discharge cycle tests were measured at a current density of 10 mA cm^−2^ at a room temperature (25 °C) on a battery testing system (CT 2001A, LanHe Instrument Technology Co., Ltd., China). The cyclic voltammetry (CV), the linear sweep voltammetry (LSV), and electrochemical impedance spectroscopy (EIS) studies were performed with an electrochemical workstation (PARSTAT 4000A, Princeton Applied Research, the United States). The CV and LSV studies were performed at a scan rate of 2 mV s^−1^ with a voltage window of 0 to ±1 V vs saturated calom elelectrode (SCE). The electrolyte is 1 M KOH with O_2_-saturated and the test temperature is 25 °C.

X-ray diffraction (XRD, Bruker D8 advanced, Germany) was used to analyze the phase composition of the electrodes after cycle testing. The scanning angle window is 10°–90°. The surface morphologies of the samples were characterized by field-emission scanning electron microscopy (SEM, JSM-7800F, with EDS (Energy Dispersive Spectroscopy)). SEM images are obtained at operating voltage of 15 kV. The concentration of carbonate ion (CO_3_^2–^) in the electrolyte was obtained by chemical titration.

## 3 Results and discussion

### 3.1 Galvanostatic discharge performance

**FIGURE 3:**
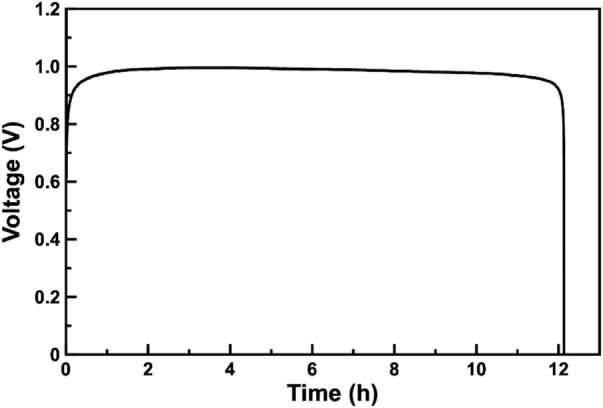
Galvanostatic discharge curve of ZAB at a constant current density of 10 mA cm^−2^.

Figure 3 presents the discharge profile of the assembled ZAB. The assembled ZAB can be galvanostatic discharged at about 1 V for about 12 h. The discharge voltage is close to the data reported in the literature (Zhong et al., 2021). The discharge capacity of the battery is 540 mAh, the specific discharge capacity is 120 mAh cm^−2^, which is sufficient for applications in electrical vehicles, grid energy storage, even some advanced electronics (Song et al., 2022).

### 3.2 Charge/discharge cycle performance

**FIGURE 4:**
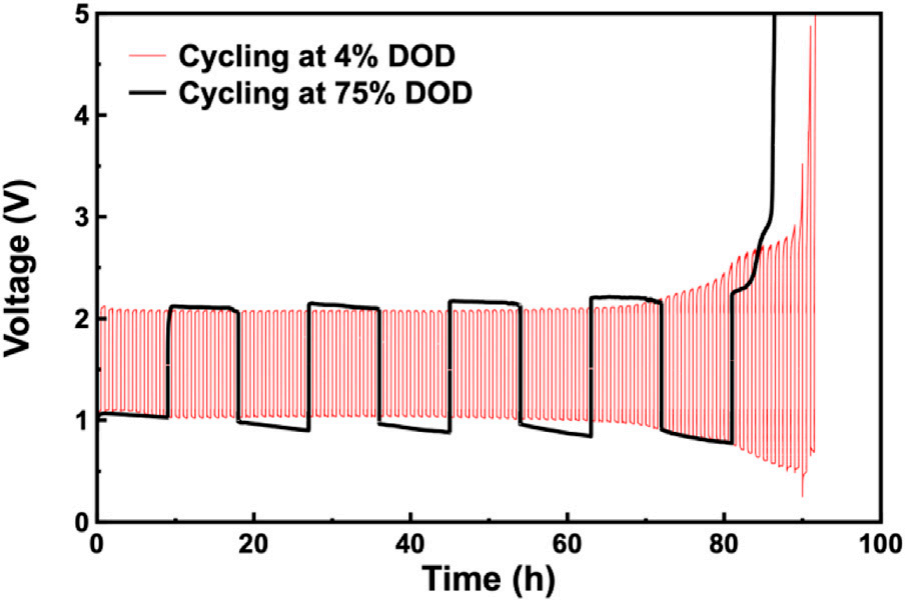
Galvanostatic charge/discharge cycle curves for assembled ZABs cycling respectively at 4% and 75% DOD, respectively.

To investigate the effect of depth of discharge (DOD) on its cycle life, two DODs were selected, the 4% shallow DOD, and the 75% deep DOD. The galvanostatic charge/discharge cycle curves are showed as in Figure 4. The cycle lives at 4% and 75% DOD were 91.64 h and 86.50 h at 91.5 and 4.5 cycles, respectively. In the shallow DOD cycling, the voltage decreased during cycling but never dropped below 1 V in the first 70 h of the cycles, after about 70 continuous cycles, the charge/discharge voltage continued to increase/decrease significantly, result in a gradually enlarged voltage difference. While a more pronounced and gradually enlarged voltage difference was observed in the deep DOD cycling. The failure for the assembled ZABs during the charging periods showed the end-of-discharge (EOD) voltage during the 4% DOD and 75% DOD cycling. The difference of cycle lives cycling at 4% and 75% DOD was only 5.61%, indicating that cycle life of ZAB is not greatly affected by the depth of discharge (DOD). The gradually enlarged voltage difference and especially the failure suggested that the side reactions, such as the hydrogen evolution, passivation and dendrite growth of Zn anode, the degradation of carbon-based Co_3_O_4_/CB air cathode and water consumption and carbonation of electrolyte, occurred during the discharge/charge processes.

### 3.3 Replacement experiments

**FIGURE 5:**
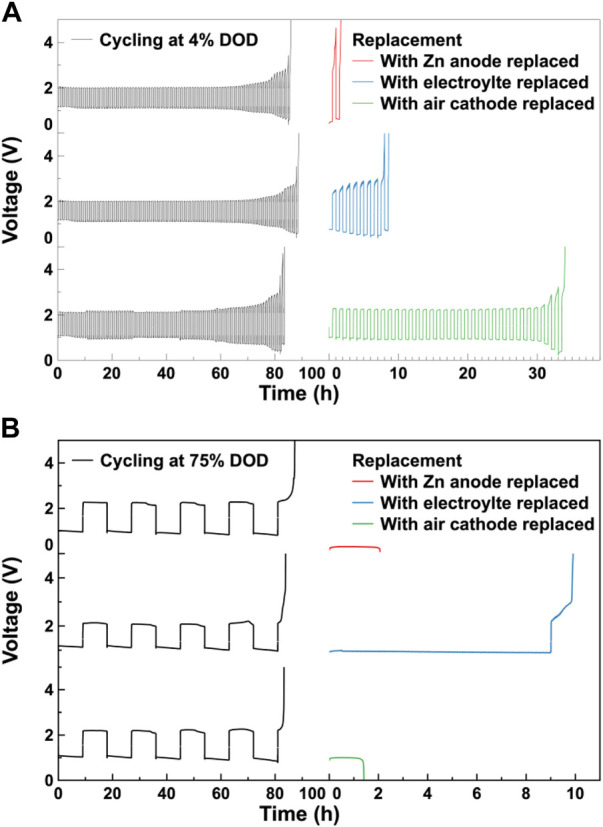
Replacement experiment. Galvanostatic charge/discharge cycle curves for ZABs and cycled ZABs with Zn anode, electrolyte and Co_3_O_4_/CB air cathode replaced, respectively at **(A)** 4% DOD and **(B)** 75% DOD, respectively.

To study the effect of each component on the cycle life of the assembled ZABs, a set of replacement experiments was conducted. After cycling, each component of the cycled ZABs, including the Zn anode, the electrolyte and the Co_3_O_4_/CB air cathode, was replaced by a fresh one, respectively. Then, the replaced ZABs were subjected to the charge/discharge cycle test under the same cycling conditions. The results of the replacement experiments are shown in Figures 5A, B. The galvanostatic charge/discharge cycle curves for ZABs cycling at 4% DOD and 75% DOD match the results in Figure 4. In the 4% DOD cycle, the cycle life of the battery with replaced Co_3_O_4_/CB air cathode is the longest, which can cycle 34 h, the cycle life of the ZABs with replaced electrolyte is about 10 h, and the ZAB with replaced Zn anode has the shortest cycle life of 1.5 h. This indicates that in the shallow charge/discharge cycle, the air electrode has the greatest impact on the cycle life of the assembled ZABs, followed by the electrolyte, and the Zn anode has the least. In the 75% DOD cycle, the cycle life of the replaced battery with electrolyte is the longest, which can cycle for 10 h. The cycled ZABs with replaced Zn anode and air electrode have cycle lives of about 2 h and 1.5 h, respectively. This indicates that in the deep charge/discharge cycle, the electrolyte has the greatest impact on the cycle life of the assembled ZABs. The limited effect of Zn anode on ZAB’s cycle life is consistent with related studies (Song et al., 2022), suggesting that Zn is in relative excess in current battery molds. Furthermore, it should be noted that, limited by the electrolyte, the air electrode and the Zn anode affect ZAB’s cycle life to an almost equal extent, which can not be distinguished under the investigated experimental conditions. In this regard, further experiments were carried out.

**FIGURE 6:**
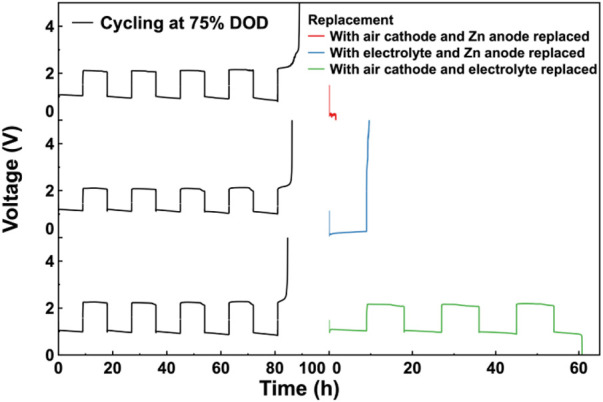
Replacement experiments. Galvanostatic charge/discharge cycle curves for ZABs and cycled ZABs with two components among Zn anode, electrolyte and Co_3_O_4_/CB air cathode replaced once in turn after cycling at 75% DOD.

To further clarify the influence of the Zn anode and Co_3_O_4_/CB air cathode on the cycle life of the assembled ZABs during a deep cycling, the experiment was performed by replacing two components once. After cycling, two components of the cycled ZABs’ components were replaced by fresh ones once. Then, the replaced ZABs were subjected to the charge/discharge cycle test under the same cycling conditions. The results were showed as Figure 6. The results of the 2-component replacement experiments cycling at 75% DOD shows that the replaced ZAB with Co_3_O_4_/CB air cathode and Zn anode has the shortest cycle life of 2 h, indicating that the electrolyte has the greatest impact on the cycle life of the assembled ZABs, which is consistent with the results of Figure 5B. The replaced ZAB with electrolyte and Zn anode has a longer cycle life of 9 h, and the replaced ZAB with electrolyte and Co_3_O_4_/CB air cathode has the longest cycle life of more than 60 h, which is close to a new assembled ZAB. It can be concluded that Co_3_O_4_/CB air cathode has a greater impact on cycle life of the assembled ZAB than the Zn anode.

### 3.4 Morphological and structure characterization

After clarifying the influence of each component on the cycle life of the battery, the failed batteries were disassembled and characterized to determine the failure mechanism further.

#### 3.4.1 Characterization of cycled Zn anode

**FIGURE 7:**
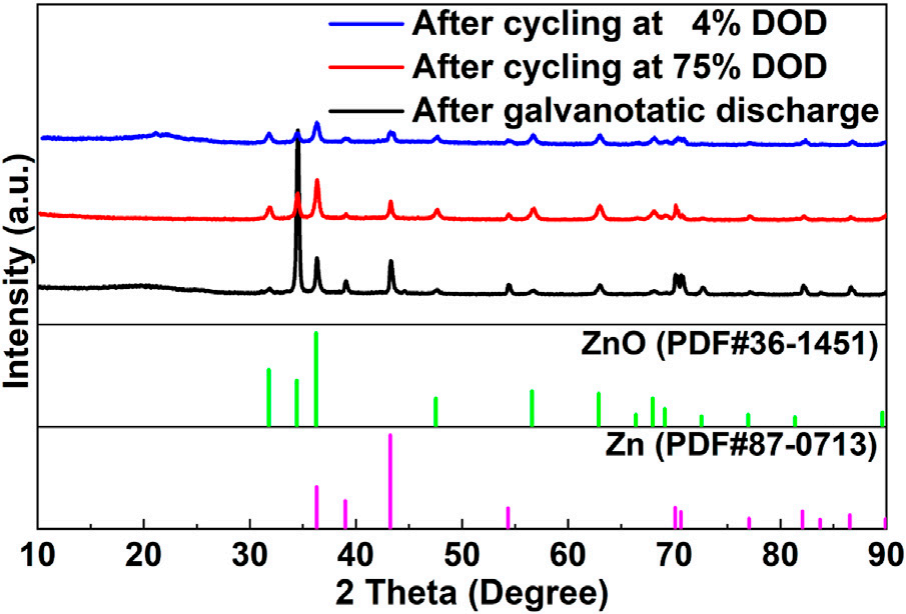
XRD results of the Zn anodes after galvanostatic discharge and cycling, respectively.

Figure 7 shows the XRD patterns of the cycled zinc anodes after galvanostatic discharge, including 4% DOD and 75% DOD. It can be observed that under different discharging/cycling conditions, the XRD patterns of the cycled Zn anodes do not show significant change. The observed peaks matched quite well with the values of Zn when compared with JCPDS No. 87–0,713 at 2θ = 36.3°, 39.0°, 43.2°, 54.3°, 70.1°, 70.6°, 82.1° and 86.5°. Besides, the peaks detected at 31.8°, 34.4°, 36.3°, 47.5°, 56.6°, 62.9°, 68.0°, 72.6° and 77.0° are assigned to the ZnO (JCPDS No. 36–1,451). The XRD profiles mentioned above demonstrate that ZnO would be formed on the anode surface after discharge. The presence of ZnO provides evidences for corrosion and passivation on the surface of Zn anodes in the galvanostatic discharge and cycling processes. The relative reactions are described as follows:

Hydrogen evolution reaction (HER) (Yi et al., 2018):

$${\text{Zn}+2\;\text{OH}}^{-}\to {\text{ZnO}+H}_{2}O+2\;e^{-}$$ (1)

$$2\;H_{2}O+2\;e^{-}\to 2\;{\text{OH}}^{-}{+H}_{2}$$ (2)

Passivation reaction (Zhou et al., 2019):

$$\text{Zn}+2\;{\text{OH}}^{-}\to \text{Zn}{\left(\text{OH}\right)}_{2}+2\;e^{-}$$ (3)

$$\text{Zn}{\left(\text{OH}\right)}_{2}+2\;{\text{OH}}^{-}\to \text{Zn}{\left(\text{OH}\right)}_{4}^{2-}$$ (4)

$$\text{Zn}{\left(\text{OH}\right)}_{4}^{2-}\to \text{ZnO}{\left(s\right)}_{\text{aq}}+2\;{\text{OH}}^{-}{+H}_{2}O$$ (5)

Hydrogen generated by the corrosion of Zn anode in alkaline environment can increase the internal pressure of the battery, damaging the battery in the action of internal swelling (Yi et al., 2018; Dongmo et al., 2020). Besides, the consumption of active metal zinc caused by corrosion will lead to the attenuation of battery capacity and self-discharge. Unfortunately, hydrogen evolution inevitably occurs in both the charge and discharge progress, even when the battery is at rest. ZnO passivation is caused by the concentration polarization and decomposition of Zn(OH)2– 4 in solution. ZnO deposits on the surface of the zinc electrode to form an insulating layer during the discharge process, which is the passivation process of Zn electrode (Farmer and Webb, 1972). The discharge process is terminated due to the insulating ZnO passivation film hinder the contact between the Zn anode and the electrolyte, besides, hindering the reduction of zincate ions could limit the rechargeability of the rechargeable alkaline Zn–air battery. (Zhu et al., 1998; Ghavami et al., 2007; Wu et al., 2018).

**FIGURE 8:**
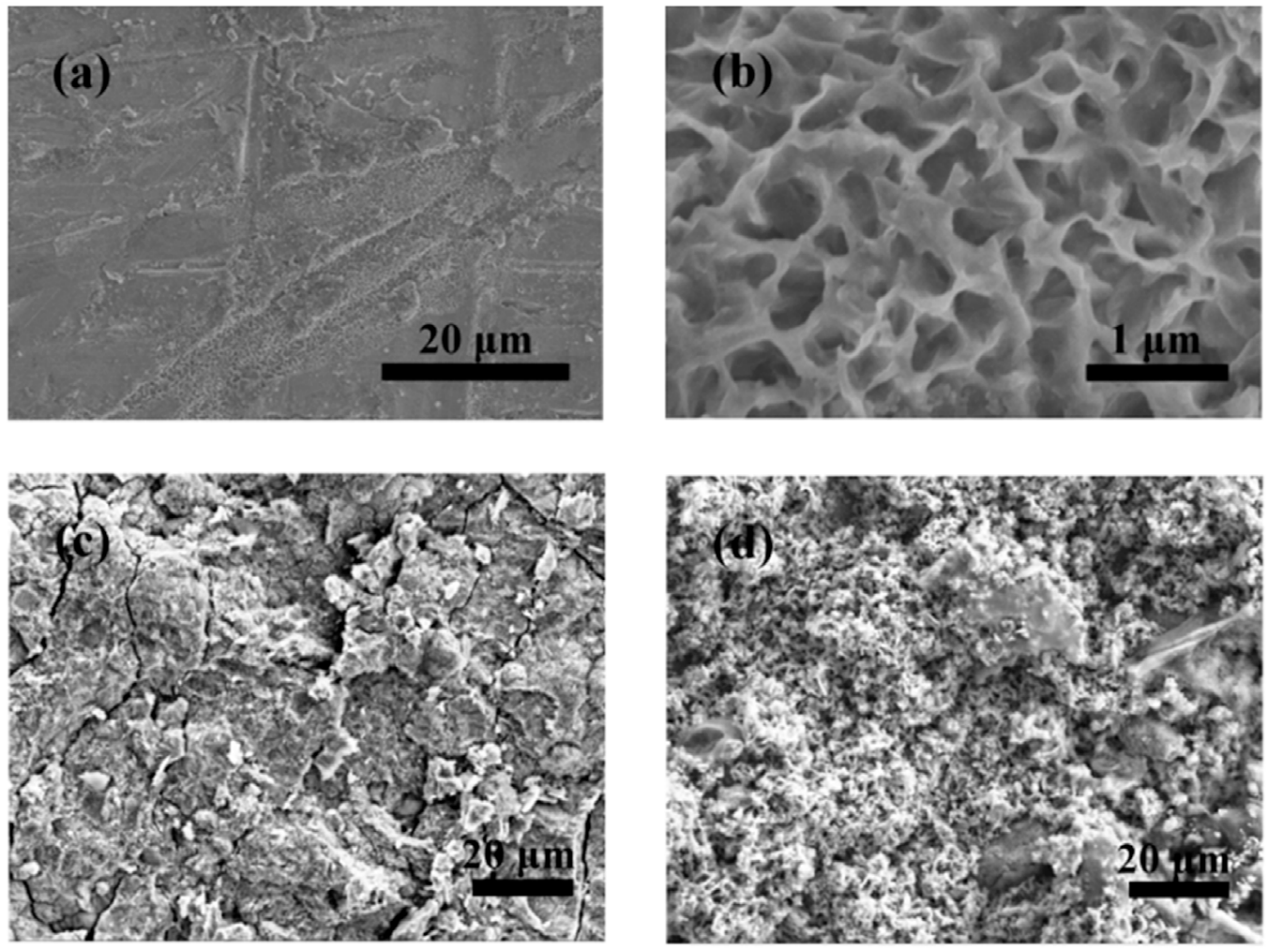
Structural characterization of the Zn anodes after galvanostatic discharge and cycling, respectively. **(A)** Low-magnification and **(B)** high-magnification SEM images of the Zn anodes after galvanostatic discharge. SEM images of Zn anodes after cycling at **(C)** 4% DOD and **(D)** 75% DOD.

Figure 8 shows the morphological changes of the Zn anodes after discharging. As shown in Figures 8A, B, after galvanostatic discharge, the surface of the Zn anode is slightly corroded, which presents a 2-dimensional honeycomb-like structure morphology. As illustrated in Figure 8C, corrosion spots can be observed on the entire surface of the Zn anode, after cycling at 4% DOD. And after cycling at 75% DOD, the surface of the Zn anode has changed from a dense structure to a porous structure as in Figure 8D.

Supplementary Figures S1, S2 show the elements mapping of Zn anodes after cycling at 4% and 75% DOD, respectively. The corresponding element contents of Zn anodes verified by EDS under the same conditions as above mentioned have been listed in Supplementary Tables S1,S2, respectively. O element observed at the surface of Zn anodes, suggesting the existence of ZnO on its surface after cycling as shown in Supplementary Figures S1, S2. ZnO layer forms because zinc was oxidized in the discharging progress (Beverskog and Puigdomenech, 1997). ZnO deposited on the Zn surface hinders the diffusion and exchange of the reactants and products in the solution, terminating the battery reactions and leading to a shortened discharge life (Thangavel et al., 2020).

#### 3.4.2 Characterization of cycled Co_3_O_4_/CB air cathodes

**FIGURE 9:**
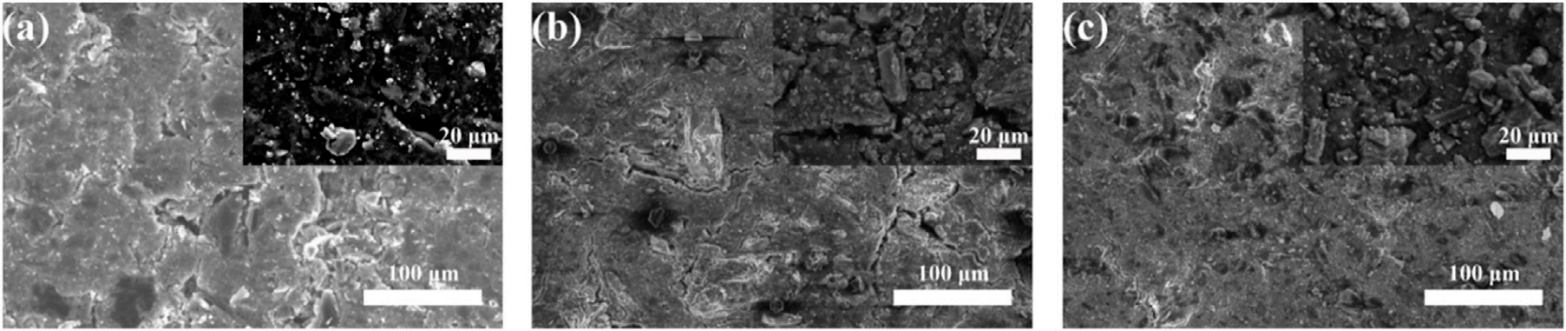
SEM images of the Co_3_O_4_/CB air cathodes before and after cycling, respectively, with corresponding high-magnification SEM images in the inset. **(A)** SEM image of the Co_3_O_4_/CB air cathodes before cycling. SEM images of Co_3_O_4_/CB air cathodes after cycling at **(B)** 4% DOD, **(C)** 75% DOD.

Figure 9 shows the morphological changes of Co_3_O_4_/CB air cathode before and after cycling, respectively. In Figure 9A, the distribution of particles is relatively uniform. As shown in Figures 9B, C, there are pieces of crystal materials on the surface of Co_3_O_4_ with different size. The crystal materials in Figure 9B are more obvious than that in Figure 9C. Supplementary Figures S3–S5 and Supplementary Tables S3–S5 show the relative elements mapping and element contents verified by EDS, respectively. As shown in Supplementary Figures S3–S5, the K and Zn elements were observed on the surfaces of Co_3_O_4_/CB air cathodes. K element in Supplementary Figures S4, S5 have distributions consistent with C and O elements accumulation, Co. and Zn elements appear less frequently than K element. This indicates that the active sites of Co_3_O_4_/CB air cathode are covered by K_2_CO_3_, and a smaller amount of Zn element is deposited on the surface of Co_3_O_4_/CB air cathode. Based on Supplementary Tables S3–S5, it can be deduced that blockage of active sites on the surface of Co_3_O_4_/CB air cathode by lamellar crystals K_2_CO_3_ after cycling at 4% DOD is more serious. After cycling, the reduction of C element on the Co_3_O_4_/CB air cathode surface indicates that C element is oxidized at the high discharge voltage, that is, carbon corrosion of Co_3_O_4_/CB air cathode. The oxidized carbon during carbon corrosion is also one of the main carbon sources for the carbonation of potassium hydroxide. The relative reactions are shown as (Chang et al., 2021; He et al., 2022):

$${C+4\;\text{OH}}^{-}{=\text{CO}}_{2}+2\;H_{2}O+4\;e^{-}$$ (6)

$$2\;{\text{KOH}+\text{CO}}_{2}{=K}_{2}{\text{CO}}_{3\_}{+H}_{2}O$$ (7)

Another important carbon source for ZABs is the diffusion through Co_3_O_4_/CB air cathode of CO_2_ in the air together with O_2_ (Chang et al., 2021).

**FIGURE 10:**
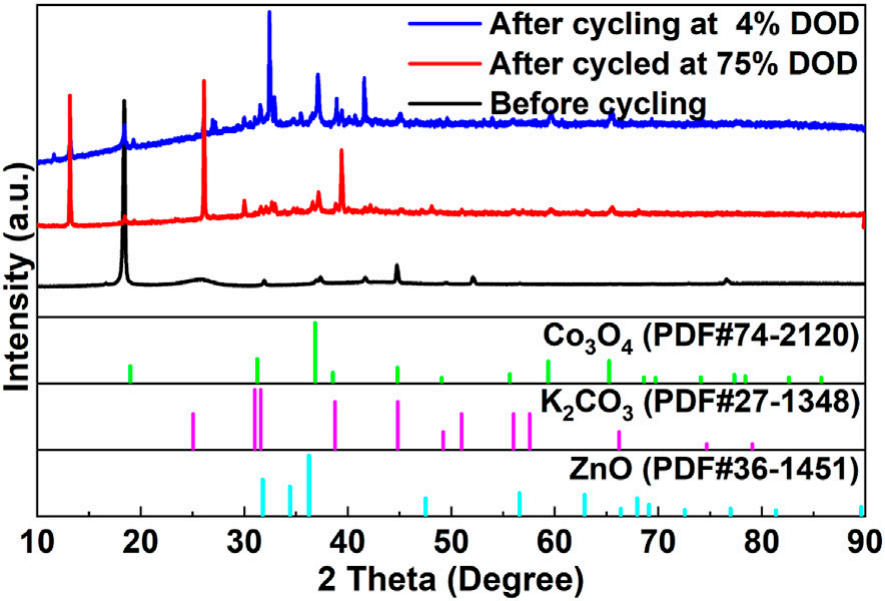
XRD results of the Co_3_O_4_/CB air cathodes before and after cycling, respectively.

Figure 10 shows the XRD patterns of the cycled Co_3_O_4_/CB air cathodes before cycling, after cycling at 4% DOD and 75% DOD, respectively. Under different cycling conditions, the XRD patterns of the cycled Co_3_O_4_/CB air cathodes do not show significant change. The observed peaks are consistent with Co_3_O_4_ (JCPDS No. 74–2,120) at 2θ = 19.0°, 31.3°, 36.8° 44.8°, 59.3° and 65.2°. Besides, the peaks detected at 25.1°, 31.0°, 31.6°, 38.8°and 44.9° are assigned to the K_2_CO_3_ (JCPDS No. 27–1,348). The peaks of zinc oxide (ZnO) can be clearly observed at 2θ = 31.8°, 34.4°, 36.3°, 47.5° and 56.60° (JCPDS No. 36–1,451). The XRD results demonstrate that K_2_CO_3_ and ZnO are not observed existing before cycling, but forms on the cathode surface after cycling, indicating the precipitation of K_2_CO_3_ and the deposition of ZnO occur during the cycling, which can lead to the active sites on the surface of the Co_3_O_4_/CB air cathode being shielded (Chang et al., 2021; Zhong et al., 2021; He et al., 2022).

**FIGURE 11:**
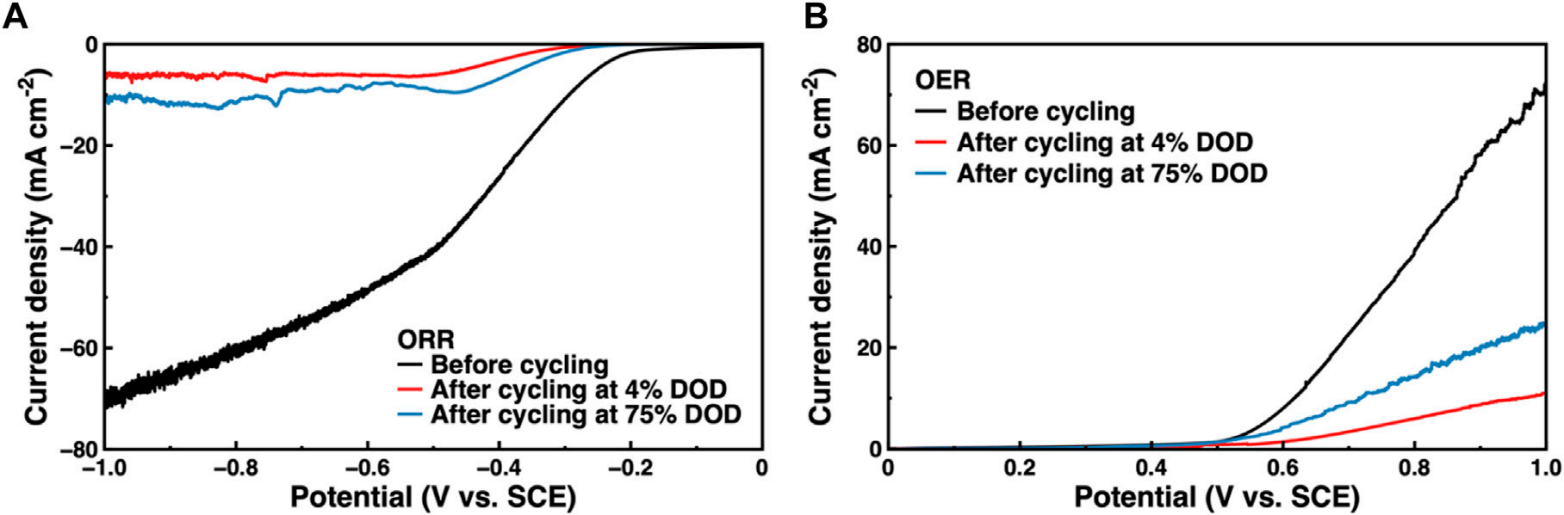
ORR/OER of Co_3_O_4_/CB air cathodes before and after cycling. **(A)** ORR results, and **(B)** OER results.

The ORR and OER of the Co_3_O_4_/CB air cathode were tested to further study the causes of Co_3_O_4_/CB air cathode failure. The ORR/OER results are illustrated in Figure 11. The ORR and OER performance of the Co_3_O_4_/CB air cathode decreased significantly after cycling. The attenuations of ORR/OER’s potential value after cycling at 4% DOD are more than that cycling at 75% DOD. The environment of the Co_3_O_4_/CB air cathode in a 1 M KOH with O_2_-saturated solution is not exactly the same with that of the Co_3_O_4_/CB air cathode in the assembled ZAB. When the Co_3_O_4_/CB air cathode is working in the assembled ZAB, O_2_ continuously enters through the hydrophobic layer. Considering the different test environment, the ORR/OER result can qualitatively explain the performance decreases of the Co_3_O_4_/CB air cathode. The gradually active sites covered by precipitated K_2_CO_3_ and the deposited ZnO result in a reduction of effective reaction area, indicating that the effective reaction area of active sites in the Co_3_O_4_/CB air cathode after cycling at 4% DOD decreases more than that after cycling at 75% DOD cycles (Zhong et al., 2021; He et al., 2022).

#### 3.4.3 Concentration of CO_3_^2–^ after galvanostatic discharging/cycling

**FIGURE 12:**
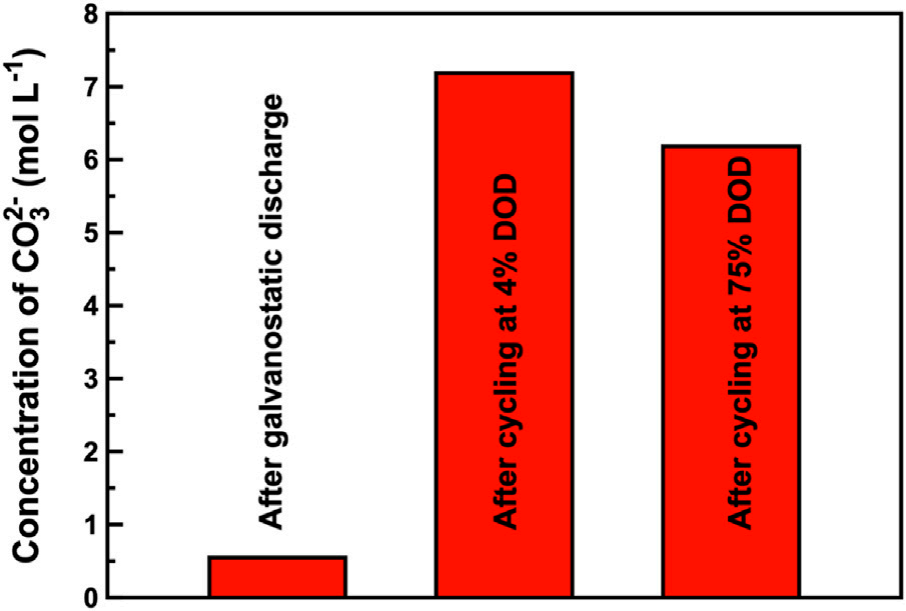
Concentration of CO_3_^2–^ in electrolyte after galvanostatic discharge and cycling, respectively.

To study the degree of carbonation of the electrolyte after discharging/cycling, chemical titration was performed to the study concentration of carbonate ion (CO_3_^2–^) in the electrolyte. Figure 12 shows the concentration of CO_3_^2–^ in electrolyte after galvanostatic discharge or cycling. After the galvanostatic discharge process, the concentration of CO_3_^2–^ in electrolyte becomes 0.547 mol L^−1^, which is the lowest. The measured concentration is 7.186 mol L^−1^ after cycling at 4% DOD, while it changes into 6.182 mol L^−1^ after cycling at 75% DOD. The results are close to the related research (Zhong et al., 2021; He et al., 2022; Song et al., 2022). The difference of CO_3_^2–^ concentration is basically consistent with the operating time difference of the assembled ZAB during the discharging or cycling process. The two main carbon sources that cause carbonation of the electrolyte are the continuously oxidized carbon material in the Co_3_O_4_/CB air cathode and the CO_2_ that enters the ZAB with the continuous flow of O_2_ through the Co_3_O_4_/CB air cathode, respectively (Chang et al., 2021; Zhong et al., 2021; He et al., 2022; Song et al., 2022). Besides, evaporation of water exacerbate carbonation of the electrolyte, increasing the concentration of K_2_CO_3_ in the electrolyte (Yang and kim, 2019). Continuous increases of partial K_2_CO_3_ concentration led to the increased concentration polarization, more K_2_CO_3_ precipitated out of the electrolyte and adhered to the electrodes’ surface, hindering the interface reactions between the electrodes and the electrolyte, and affecting the cycle life of ZAB.

## 4 Conclusion

In order to explore the failure mechanism of zinc-air batteries at different depths of discharge, we proposed a ZAB mold and assembled a rechargeable ZAB to perform charge-discharge cycle tests at different depths of discharge (DOD). And then, we characterized and electrochemically tested the failed battery components. It can be found that, for an assembled ZAB, in the shallow cycle (cycled at 4% DOD), the influence of each component on ZAB’s cycle life is in order of Co_3_O_4_/CB air cathode, electrolyte and Zn anode. The coverage of active site in the Co_3_O_4_/CB air cathode has the greatest impact on the cycle life of the battery, which is caused by the coverage of K_2_CO_3_ on the surface of Co_3_O_4_/CB air cathode. The K_2_CO_3_ is precipitated by the carbonation of the electrolyte. Meanwhile, in the deep cycle (cycled at 75% DOD), the order that affects ZAB’s cycle life is the electrolyte, the Co_3_O_4_/CB air cathode, and the Zn anode. The carbonation of the electrolyte becomes the most important bottleneck factor affecting ZAB’s cycle life in the deep cycle.

As a conclusion, the development of high-stability, high-capacity, and high-efficiency cathode materials is also crucial for the application of AZBs. Although efforts have been made to explore different cathode materials for different AZBs, it is still a challenge to design cathode materials that fully meet the requirements of AZBs. The development of efficient cathode catalysts is crucial to realize high-performance Zn-air batteries. So solving the carbonation of the electrolyte in ZAB will effectively improve the cycle life of ZAB, which turns to be the key research content of ZAB’s large-scale commercial use.

## Data Availability

The original contributions presented in the study are included in the article/Supplementary Material, further inquiries can be directed to the corresponding author.
