# Supplementary material for: Study on failure mechanism on rechargeable alkaline zinc–Air battery during charge/discharge cycles at different depths of discharge
Source: Front Chem. 2023 Jan 20;11:1121215. doi: 10.3389/fchem.2023.1121215 (PMC9895414; doi:10.3389/fchem.2023.1121215)
Supplement: Supplementary file 1 [file DataSheet1.docx]

**Supporting Information**

**Study on Failure Mechanism on Rechargeable Alkaline Zinc–Air Battery during Charge/discharge Cycles at Different Depths of Discharge**

**Donghao Zhang^1^, Wenbin Hu^1,2,3,*^**

^1^ Key Laboratory of Advanced Ceramics and Machining Technology (Ministry of Education), School of Materials Science and Engineering, Tianjin University, No. 135 Yaguan Road, Jinnan District, Tianjin 300072, China.

^2^ Tianjin Key Laboratory of Composite and Functional Materials, School of Materials Science and Engineering, Tianjin University, No. 135 Yaguan Road, Jinnan District, Tianjin 300072, China.

^3^ Joint School of National University of Singapore and Tianjin University, International Campus of Tianjin University, Binhai New City, Fuzhou 350207, China.

*** Correspondence:**

Wenbin Hu

wbhu@tju.edu.cn

**Keywords:** **rechargeable alkaline zinc–air battery, cycle life, depths of discharge, corrosion, carbonation.**


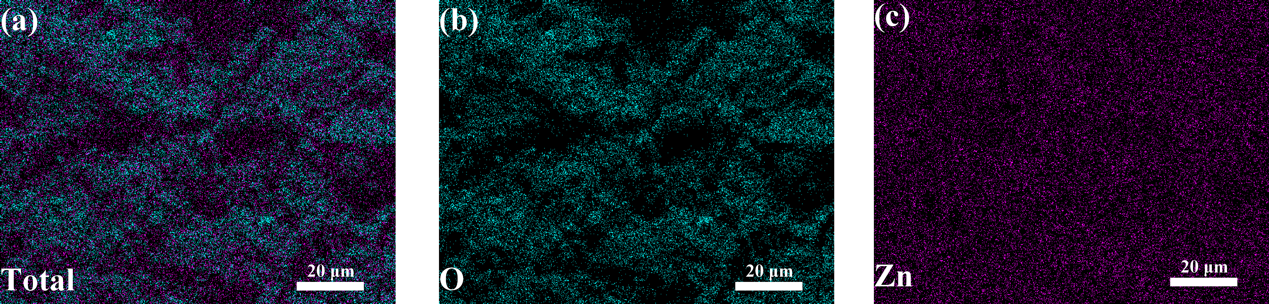


**Fig S1.** Element mapping of Zn anodes after galvanostatic charge/discharge cycling at 4% DOD. a) Total, b) O, c) Zn, respectively.


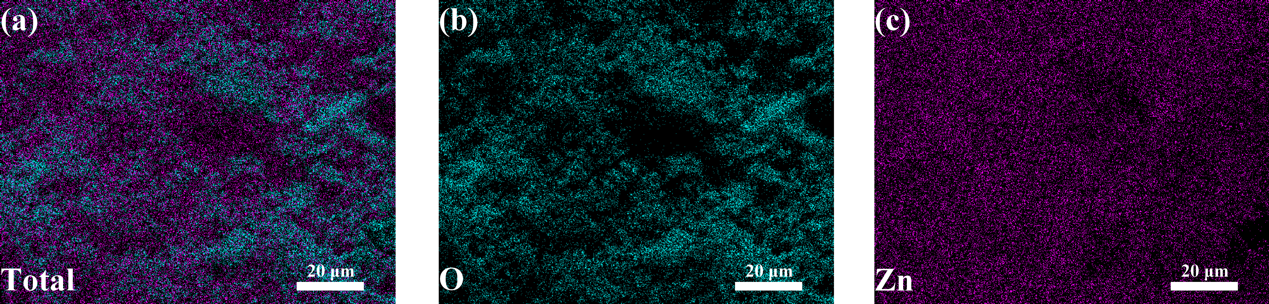


**Fig S2.** Element mapping of Zn anodes after galvanostatic charge/discharge cycling at 75% DOD. a) Total, b) O, c) Zn, respectively.

**Table S1.** EDS results of Zn anodes after galvanostatic charge/discharge cycling at 4% DOD

| Element | wt% | At% |
| --- | --- | --- |
| O | 13.44 | 38.82 |
| Zn | 86.56 | 61.18 |
| Total | 100.00 | 100.00 |

**Table S2.** EDS results of Zn anodes after galvanostatic charge/discharge cycling at 75% DOD

| Element | wt% | At% |
| --- | --- | --- |
| O | 12.83 | 37.56 |
| Zn | 87.17 | 62.44 |
| Total | 100.00 | 100.00 |


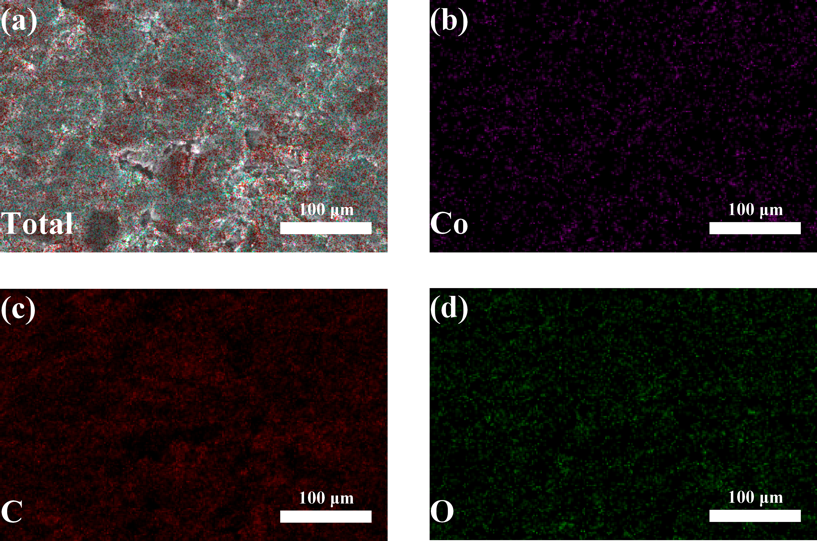


**Figure S3.** Element mapping of Co_3_O_4_/CB air cathodes before cycling. a) Total, b) Co, c) C and d) O, respectively.


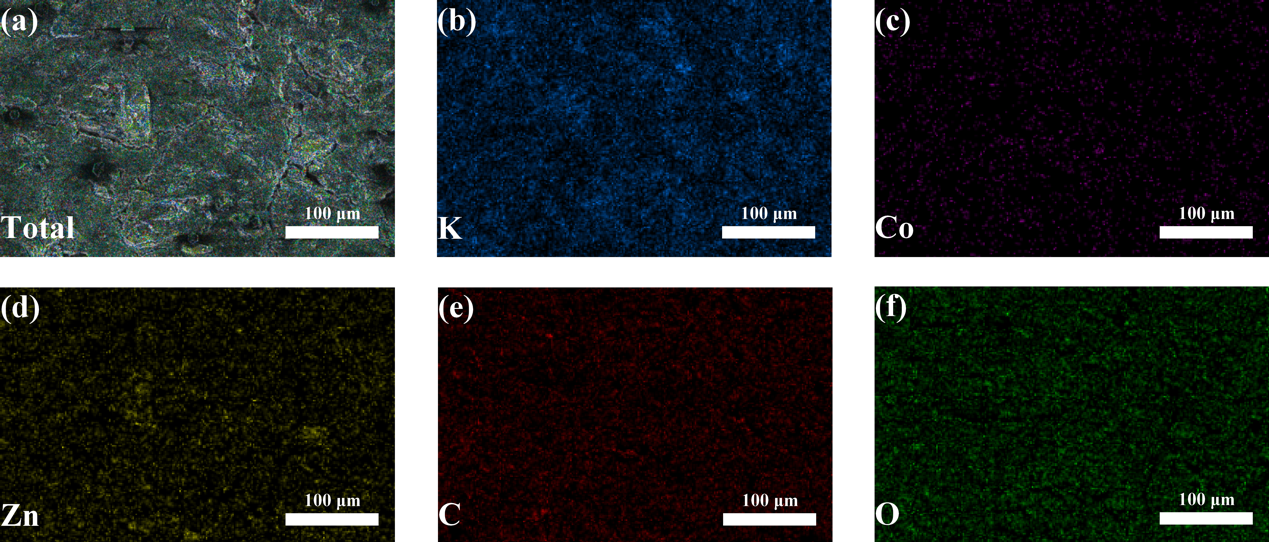


**Figure S4.** Element mapping of Co_3_O_4_/CB air cathodes after cycling at 4% DOD. a) Total, b) K, c) Co, d) Zn, e) C and f) O, respectively.


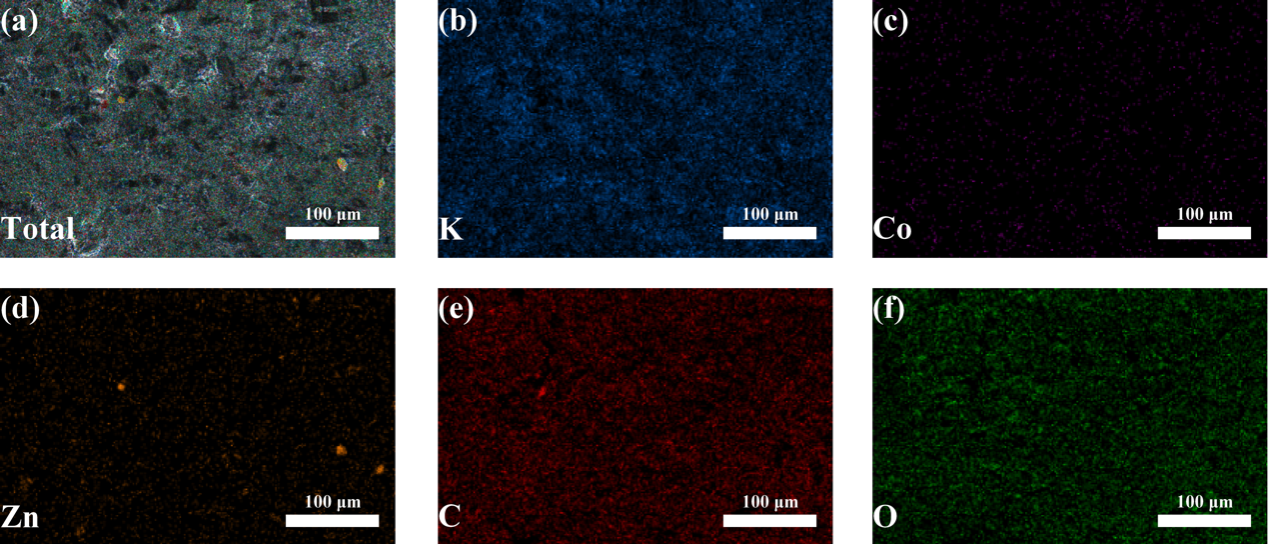


**Figure S5.** Element mapping of Co_3_O_4_/CB air cathodes after cycling at 75% DOD. a) Total, b) K, c) Co, d) Zn, e) C and e) O, respectively.

**Table S3.** EDS results of Co_3_O_4_/CB air cathodes before cycling

| Element | Wt % | At% |
| --- | --- | --- |
| C | 75.10 | 86.30 |
| O | 12.52 | 10.80 |
| Co | 12.38 | 2.90 |
| Total | 100.00 | 100.00 |

**Table S4.** EDS results of Co_3_O_4_/CB air cathodes after cycling at 4% DOD

| Element | Wt % | At% |
| --- | --- | --- |
| C | 28.41 | 44.58 |
| O | 34.36 | 40.47 |
| K | 21.00 | 10.12 |
| Co | 4.64 | 1.49 |
| Zn | 11.58 | 3.34 |
| Total | 100.00 | 100.00 |

**Table S5.** EDS results of Co_3_O_4_/CB air cathodes after cycling at 75% DOD

| Element | Wt % | At% |
| --- | --- | --- |
| C | 32.18 | 46.60 |
| O | 37.73 | 41.02 |
| K | 24.11 | 10.72 |
| Zn | 3.76 | 1.00 |
| Co | 2.23 | 0.66 |
| Total | 100.00 | 100.00 |
